# Results of the radiation dose of head, body and tail of hippocampus in nasopharyngeal carcinoma patients treated with intensity modulated radiotherapy

**DOI:** 10.1038/s41598-018-23127-6

**Published:** 2018-04-04

**Authors:** Sun Zong-wen, Shi lei, Li Qinglin, Kong yue, Du feng-lei, Xie Tie-ming, Hua Yong-hong, Hu Qiao-ying, Chen Xiao-zhong, Chen Yuan-yuan, Chen Ming

**Affiliations:** 1 Department of Oncology, Jining No.1 People’s Hospital, Jining, 272100 China; 2 Radiology Department, Zhejiang Cancer Hospital, Hangzhou, 310022 China; 3 Pharmacy Department, Zhejiang Cancer Hospital, Hangzhou, 310022 China; 4 Department of Radiation Oncology, Zhejiang Key Lab of Radiation Oncology, Zhejiang Cancer Hospital, Hangzhou, 310022 China

## Abstract

This study is to analyze the radiation dose of head, body and tail of hippocampus (HC) of nasopharyngeal carcinoma (NPC) patients treated with intensity modulated radiotherapy (IMRT). Evaluate cognitive function of patients with Wechsler adult intelligence scale-Chinese revised (WAIS-CR). HC were segmented into HC head (HH), HC body (HB) and HC tail (HT) and the indexes were then analyzed. WAIS-CR was tested before and 3months after radiotherapy. The mean radiation dose of left and right HC was (1147 ± 976)cGy, (1011 ± 602)cGy respectively. The radiation dose and the volume exposed in different dose of HH, HB and HT decreased in turn. For 5 patients, before and after radiotherapy, the regular-order score was 8.60 ± 1.34, 8.0 ± 1.00 (P = 0.43), while the reverse-order score was 5.80 ± 0.84, 5.20 ± 0.84 (P = 0.07). The radiation dose of HH, HB and HT was different, and the radiation dose of HH was the highest, which should be emphasized especially.

## Introduction

Nasopharyngeal carcinoma (NPC) is common in Southern China. Intensity modulated radiotherapy (IMRT) is frequently used in NPC radiotherapy. Compared with two-dimensional radiotherapy and three-dimensional conformal radiotherapy, there is obvious dosimetric advantages in IMRT. While the target of tumor is covered highly conformal, the exposed dose of the organs at risk (OARs) minimize as low as possible. However the normal tissues surrounding the target that has not been concerned may be irradiated with relatively a lot of dose^1^. Hippocampus (HC) is closely related with intelligence and memory, and receives relatively a lot of radiation dose in the treatment of NPC patients^2^. The result of RTOG 0933^3^ suggests that HC sparing during brain radiotherapy can alleviate memory impairment.

HC can be segmented into three parts: head (HH), body (HB) and tail (HT) according to anatomic structure. These three parts play different roles in Alzheimer’s disease^4,5^. At present, there is no study focusing on the radiation dose of the three parts of HC in NPC. This study makes a preliminary analysis of the radiation dose of the three parts.

## Material and Methods

### Patient clinical data

10 NPC patients treated from August 2015 to July 2016 in Zhejiang Cancer Hospital were enrolled. All patients were pathologically confirmed as NPC, 6 male cases, 4 female cases, 22–57 years old. According to the UICC 2010 stage of nasopharyngeal carcinoma, stage of the patients was T3-4N1-3M0, III-IVB. All 10 patients received 2–3 cycles of TP or PF (docetaxel + cisplatin/nadeplatin, fluorouracil + cisplatin/nadeplatin) induction chemotherapy, followed by IMRT and cisplatin/nadeplatin concurrent chemotherapy. Informed consent forms were signed by all patients. The ethics institutional review board of Zhejiang Cancer Hospital approved the protocols for data collection and analyses. All the methods described here were performed in accordance with the relevant guidelines and regulations.

### Immobilization and scan

The patients were immobilized by head neck shoulder thermoplastic mask at comfortable position. CT-sim scan was completed with Philips Brilliance CT or GE Light Speed, and scan layer thickness was 3 mm. After CT-sim scan, MRI scan was obtained in the same posture and immobilization mode with SIEMENS Verio 3.0 T. The parameters of MRI scan were as follow: layer thickness 1.5 mm, layer space 0.45 mm, field of view (FOV) = 230 × 230 mm, TR 1440 ms, TE 11 ms, flip angle (FA) 150 degree, scan matrix 320 × 320, the rotation angle 90 degree, voxel size = 1.0 × 0.7 × 1.5 mm^3^. CT-sim and MRI scan images were transmitted into RayStation4.0 v (RaySearch Laboratories AB), then the CT and MRI images were fused and reconstructed for target and OARs delineation.

### Target delineation and dose constraint

#### Target definition and prescription dose of nasopharynx and neck

PGTVnx + rn was the irradiated target of primary nasopharyngeal tumor and retropharyngeal lymph node, prescription dose 7040 cGy/32 F. PTV1 was the irradiated target of high-risk area, prescription dose 6080 cGy/32 F. PTV2 was the irradiated target of low-risk area including the neck lymphatic drainage area, prescription dose of 5440 cGy/32 F. PTVnd was the irradiated target of cervical lymph node, prescription dose 6400 cGy/32 F.

#### Dose constraint

At least 95% PTV was covered by the prescription dose. The volume of PTV receiving more than 110% of the prescription dose did not exceed 20%, and that receiving less than 93% of the prescription dose did not exceed 1%. Dose constraint of OARs was as follow: irradiated dose of brain stem less than 54 Gy; the maximum irradiated dose of spinal cord less than 45 Gy; dose constraint of other OARs see in expert consensus. HC was not listed as OAR in treatment plan, and there was no dose constraint for HC in the process of planning.

### The delineation of HC and the segmentation of HH, HB and HT

**Figure 1:**
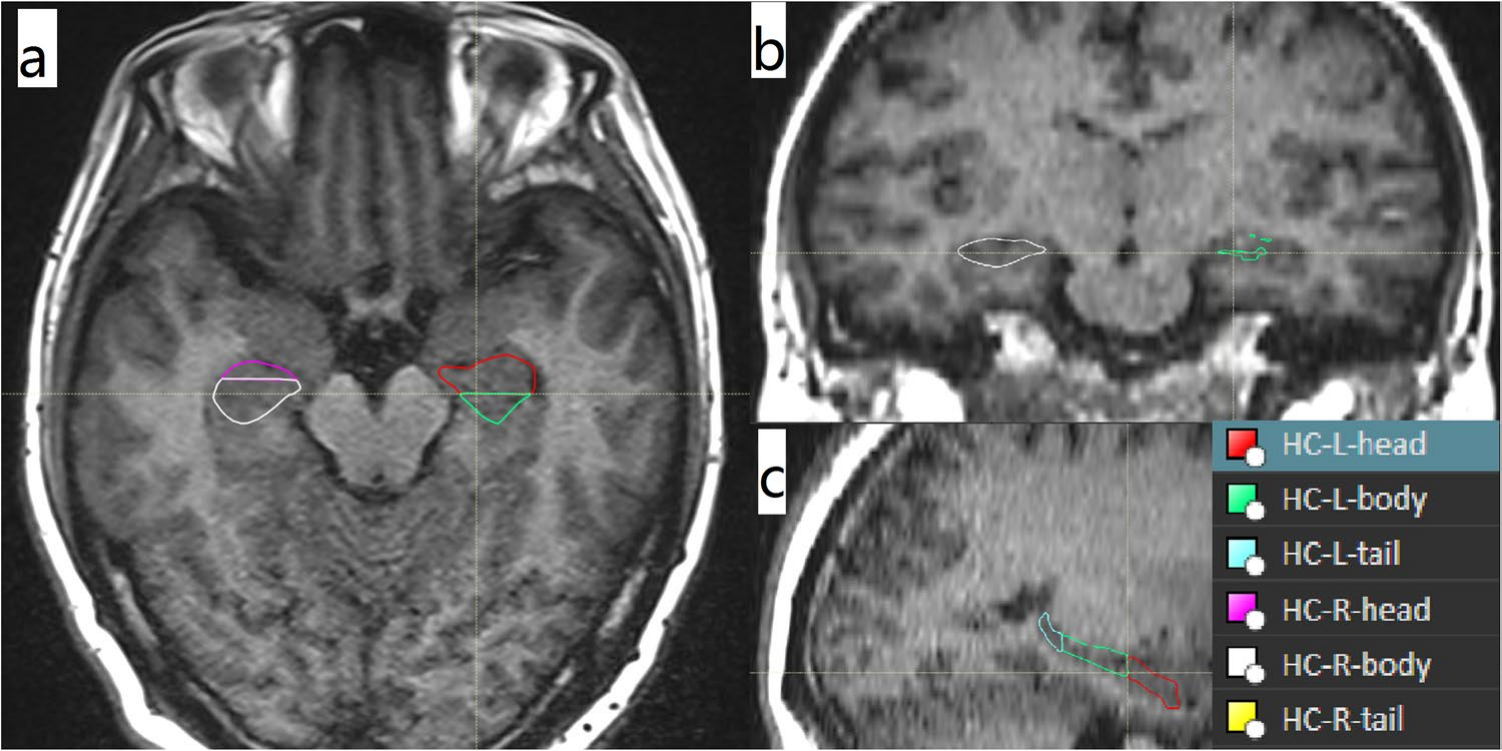
The segmentation of HC.

**Figure 2:**
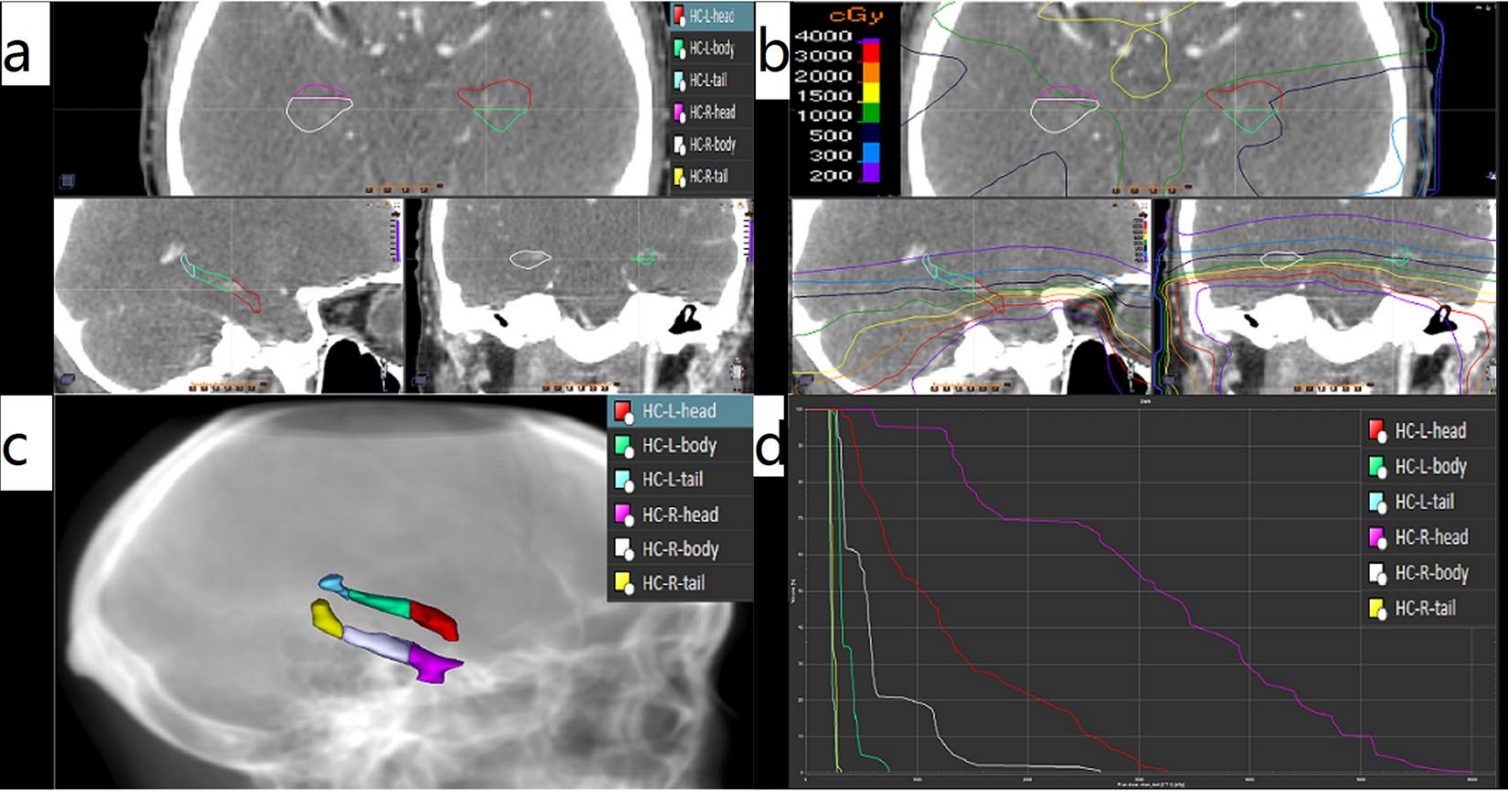
The segmentation of HC and dose distribution. (**a**) The segmentation on two dimension; (**b**) The isodose curve on two dimension; (**c**) The segmentation on three dimension; (**d**) The DVH curve.

1. The delineation of HC: According to criterion of RTOG 0933^6^, bilateral HC(HC-L, HC-R) was delineated on T1 weighted MRI images in 10 patients by two established physicians. And all delineated HC were reviewed and revised by the same senior physician.
2. The definition of HH, HB and HT: According to the anterior to posterior direction, HC was segmented into three parts: HH, HB and HT by the ratio of 35%, 45% and 20% respectively^7^.
3. The segmentation of HH, HB and HT: Fixed an image on sagittal section in which HC showed, then confirmed the total layers of the HC that showed on coronal section. With the ratio of 35%, 45% and 20%, the respective total layers of HH, HB and HT was confirmed. Meanwhile, the two sagittal layers that were based on to divide HH, HB and HT were confirmed. The two sagittal layers were showed as two lines on transverse section. Based on the two lines, HC was segmented into HH, HB and HT layer by layer. The schematic diagram was showed in Figs 1 and 2.

### Dose parameters setting

Plan designation was completed with RayStation, and then the dose parameters were collected. D_n%_ was the radiation dose of n% volume of the target organ. D_1%_ represented the maximum dose; D_99%_ represented the minimum dose; D_mean_ represented the average dose. V_n%_ represented the percentage volume of target organ that was irradiated by nGy. All data were expressed as mean ± standard deviation.

### Memory test

Wechsler adult intelligence scale-Chinese revised (WAIS-CR) was tested before and 3months after radiotherapy. All tests were carried out in quiet environment. The scale test was performed by specific physician. Only the digit span test of the WAIS-CR was used. Regular-order and reverse-order score were listed separately.

### Statistical analysis

Statistical analysis was performed with the Statistical Package for Social Sciences (SPSS, Chicago, IL) software package, version 18.0, for Windows. Single factor analysis of variance and paired T test were performed. A two-tailed P-Value less than 0.050 was considered as statistical significance in this study.

## Results

1. All 10 patients received 2–3 cycles induction chemotherapy. Effects were evaluated as PR in 9 patients (nasopharyngeal lesions close to CR in 3 patients), SD in 1 patient. Then radiotherapy and concurrent chemotherapy followed.
2. There was relatively a lot of radiation dose in bilateral HC (showed in Table 1). D_1%_, D_50%_, D_mean_ and D_99%_ of HH, HB and HT decreased in turn. The radiation dose of HH was much higher than that of HB and HT (showed in Table 2).

**Table 1:** The radiation dose of bilateral HC(cGy).

Parameters	D_1%_	D_50%_	D_mean_	D_99%_
Position				
HC-L	3049 ± 1683	935 ± 972	1147 ± 976	396 ± 480
HC-R	2923 ± 1358	832 ± 581	1011 ± 602	389 ± 403

**Table 2:** The radiation dose of HH, HB and HT.

Position		HC-L			HC-R		
Parameters		Exposed dose (cGy)	overall P value	P value between groups	Exposed dose (cGy)	overall P value	P value between groups
	HH	3160 ± 1731		0.015*	3121 ± 1410		0.001*
D_1%_	HB	1546 ± 1334	0.003	0.001^†^	1391 ± 793	0.000	0.000^†^
	HT	843 ± 1017		0.268^‡^	679 ± 714		0.131^‡^
	HH	1682 ± 1363		0.081*	1577 ± 991		0.013*
D_50%_	HB	839 ± 979	0.052	0.019^†^	713 ± 471	0.008	0.004^†^
	HT	526 ± 655		0.507^‡^	533 ± 627		0.586^‡^
	HH	1739 ± 1317		0.076*	1691 ± 942		0.006*
D_mean_	HB	890 ± 982	0.042	0.015^†^	744 ± 483	0.002	0.001^†^
	HT	547 ± 688		0.461^‡^	531 ± 603		0.503^‡^
	HH	902 ± 1016		0.288*	833 ± 575		0.067*
D_99%_	HB	533 ± 700	0.305	0.137^†^	438 ± 384	0.082	0.042^†^
	HT	380 ± 465		0.657^‡^	391 ± 407		0.822^‡^

*Represents comparison between HH and HB; ^†^Represents between HH and HT; ^‡^Represents between HB and HT.
3. The volume that was irradiated with different dose of HH, HB and HT was different. V_5_–V_50_% decreased in turn (except for the V_20_% of HC-R). The volume of HH was much higher than that of HB and HT (showed in Table 3).

**Table 3:** The volume irradiated with different radiation dose of HH, HB and HT.

Position		HC-L			HC-R		
Parameters		Volume	P value		volume	P value	
			overall P value	P value between groups		overall P value	P value between groups
	HH	80.01 ± 32.02		0.109*	81.41 ± 30.97		0.120*
V_5_%	HB	51.19 ± 44.41	0.011	0.003^†^	54.24 ± 39.83	0.005	0.001^†^
	HT	22.88 ± 39.35		0.116^‡^	20.57 ± 41.90		0.057^‡^
	HH	62.80 ± 36.20		0.020*	66.52 ± 40.02		0.034*
V_10_%	HB	24.75 ± 35.30	0.007	0.003^†^	27.48 ± 37.25	0.026	0.012^†^
	HT	11.77 ± 31.50		0.406^‡^	19.07 ± 40.26		0.635^‡^
	HH	44.53 ± 37.81		0.041*	47.86 ± 39.41		0.021*
V_15_%	HB	12.22 ± 31.13	0.052	0.030^†^	12.48 ± 27.89	0.023	0.014^†^
	HT	10.00 ± 31.62		0.884^‡^	9.79 ± 28.09		0.853^‡^
	HH	31.73 ± 33.89		0.120*	37.38 ± 36.48		0.003*
V_20_%	HB	10.33 ± 31.51	0.154	0.077^†^	**2**.**50 **±** 5**.**97**	0.005	0.006^†^
	HT	7.21 ± 22.80		0.817^‡^	**5**.**65 **±** 17**.**87**		0.768^‡^
	HH	16.31 ± 32.84		0.539*	12.92 ± 17.23		—
V_30_%	HB	9.18 ± 29.04	0.472	0.225^†^	0.00 ± 0.00	—	—
	HT	2.09 ± 6.62		0.541^‡^	0.00 ± 0.00		—
	HH	11.18 ± 27.74		0.180*	3.05 ± 9.12		—
V_40_%	HB	1.22 ± 3.87	0.255	—	0.00 ± 0.00	—	—
	HT	0.00 ± 0.00		—	0.00 ± 0.00		—
	HH	4.70 ± 12.83		0.171*	1.01 ± 3.20		—
V_50_%	HB	0.04 ± 0.13	0.281	—	0.00 ± 0.00	—	—
	HT	0.00 ± 0.00		—	0.00 ± 0.00		—

Blod number indicated the radiation dose of HT was greater than HB; ^*^Represents comparison between HH and HB; ^†^Represents between HH and HT; ^‡^Represents between HB and HT; There is no statistic analysis when mean value was 0.
4. Five patients completed WAIS-CR test. Before and after radiotherapy, the mean value of regular-order scores was 8.60 ± 1.34, 8.0 ± 1.00 (P = 0.43), and the mean value of reverse-order scores was 5.80 ± 0.84, 5.20 ± 0.84 (P = 0.07).

## Discussion

The 5 year overall survival(OS) of stage I, II, III and IVa-b NPC patients was 100%, 94.3%,83.6% and 70.5% respectively^8^. Because of so good curative effect, it is extremely important to reduce complications and improve quality of life of NPC patients. Radical radiotherapy is the main treatment for non metastatic NPC patients. The intelligence, memory and cognitive function of NPC patients decreased after radiotherapy^9,10^. In this group, 5 patients completed the WAIS-CR test. After radiotherapy, the patients decreased in the mean value of regular-order and reverse-order repetition, and the P value of reverse-order repetition was close to statistical significance (P = 0.07). This result also indicated that cognitive function impairment decreased after radiotherapy in NPC patients.

Located in the inner side of temporal horn, HC is closely related to cognitive function. In 1957, Scoville and Milner^11^ reported that the removal of HC could cause memory impairment in space and time, especially the loss of ability to form new declarative long-term memory. The severity of impairment depended on the extension of resection degree of HC. Animal experiments showed that HC played an important role in cognitive impairment caused by radiotherapy. The granular cells of the HC dentate gyrus were extremely sensitive to radiotherapy, and only 1 Gy could reduce proliferation of the cells. The neurogenesis of HC was inhibited after radiotherapy, which was closely related to cognitive impairment^12^. The mice whose HC was irradiated with 10 Gy showed cognitive impairment in Barnes maze^13^. RTOG 0933 showed that HC sparing could reduce the cognitive impairment in patients who underwent brain radiotherapy^3^. Based on RTOG0933 protocol, animal experiments also demonstrated that the cognitive impairment test of the HC sparing mice was significantly better than that of non HC sparing group^14^. Cognitive impairment aggravated significantly when the D_40%_ of bilateral HC was greater than 7.3 Gy in study of brain tumor^15^.

These results suggest that HC plays an important role in the cognitive impairment caused by radiotherapy. At present, whether Chinese 2010 nasopharyngeal carcinoma intensity modulated target expert consensus^16^ or RTOG 0615^17^, HC was not listed as OAR, and the tolerance dose of HC is not clear.

The radiation dose of HC was reported differently under the technology of IMRT without HC sparing. Khodayari *et al*.^2^ reported that the D_mean_ of HC was 3027 cGy (range 1908-4799cGy), while the D_mean_ of HC was 1518 cGy reported by Gu *et al*.^18^. And the D_mean_ was (2411 ± 239)cGy in the report of Han *et al*.^19^. In this study, the D_mean_ of HC-L and HC-R was (1147 ± 976) cGy, (1011 ± 602) cGy respectively, which was lower than those reported previously. The possible reasons were as follow: on one hand there was certain randomness in the radiation dose of HC as there was no dose constraint; on the other hand patients in this group all underwent 2–3 cycles of induction chemotherapy. Then the irradiated target volume reduced, so the radiation dose of HC reduced to some extent.

HC is a symmetry structure that goes along from anterior-inferior to posterior-superior, and can be segmented into head, body and tail. In 2002 Hackert *et al*.^7^ segmented HC into HH, HB and HT with the ratio of 35%, 45% and 20% respectively, and did related research. Study showed that dysfunction of HH played an important role in memory impairment in patients with amnesia^20^. Oral memory deficits were associated with left HH atrophy in the patients with traumatic brain injury^21^. Yakushev *et al*.^4^ found that an increase in the diffusion rate of the left HH was significantly associated with delayed verbal memory recall test (DVR), and volume decrease in the left HB and HT was associated with DVR. In the study of Alzheimer’s disease, the atrophy of the HH, HB and HT was different, and the HH, HB and HT had unique relationship with the neurocognitive function and the indexes of cerebrospinal fluid^5^.

In view of different function of HH, HB and HT, we hypothesize that the role of HH, HB and HT may be different in neurocognitive impairment caused by radiotherapy. Therefore, it is necessary to analyze the radiation dose of HH, HB and HT.

The radiation dose was significantly different in HH, HB and HT. The D_1_%, D_50_%, D_mean_ and D_99_% of HH, HB and HT decreased in turn, and the radiation dose of HH was much higher than that of HB and HT. In Fig. 2b and d, the isodose curve also showed the dose difference of HH, HB and HT. There was no overall statistic difference in D_99%_ of bilateral HC, D_50%_ of HC-L, but there was overall statistic difference in all other parameters. Comparative analysis among HH, HB and HT showed that in comparison between HH and HB, there was statistic difference in D_1%_, D_50%_, D_mean_ of HC-R, D_1%_ of HC-L; in comparison between HH and HT, all parameters reached statistic difference except for D_99%_ of HC-L; as for the comparison between HB and HT, there was no statistic difference in D_1%_, D_50%_, D_mean_, D_99%_. These results indicated that the differences between HH and HT, HH and HB, HB and HT decreased in turn. There was no statistic difference in some parameters, but the difference in mean value was obvious. The difference was not statistically significant possibly because of the small sample size.

The volume irradiated with different radiation dose of HH, HB and HT was different, and the volume of HH was much higher than that of the HB and HT. The V_5_%–V_50_% of HH, HB and HT decreased in turn (except for the V_20_% of HC-R). There was overall statistic difference in V_5_% and V_10_% of HC-L, V_5_% to V_20_% of HC-R, and there was almost statistic difference in V_15_% of HC-L (p = 0.052). Comparative analysis among HH, HB and HT showed that in comparison between HH and HB, there was statistic difference in V_10_% and V_15_% of the HC-L, and V_10_%–V_20_% of HC-R; in comparison between HH and HT, there was statistic difference in V_5_%–V_15_% of HC-L and V_5_%–V_20_% of HC-R; as for comparison between HB and HT, there was no statistic difference in all parameters of bilateral HC. From the beginning of V_30_%, the HB and HT of HC-R was 0, while V_40_% of HT-L was 0. The volume irradiated with different dose also showed that the difference between the HH and HT, HH and HB, HB and HT decreased in turn. There was no statistic difference in some parameters, but the difference in mean value was also obvious. The difference was not statistically significant possibly because of the small sample size.

**Figure 3:**
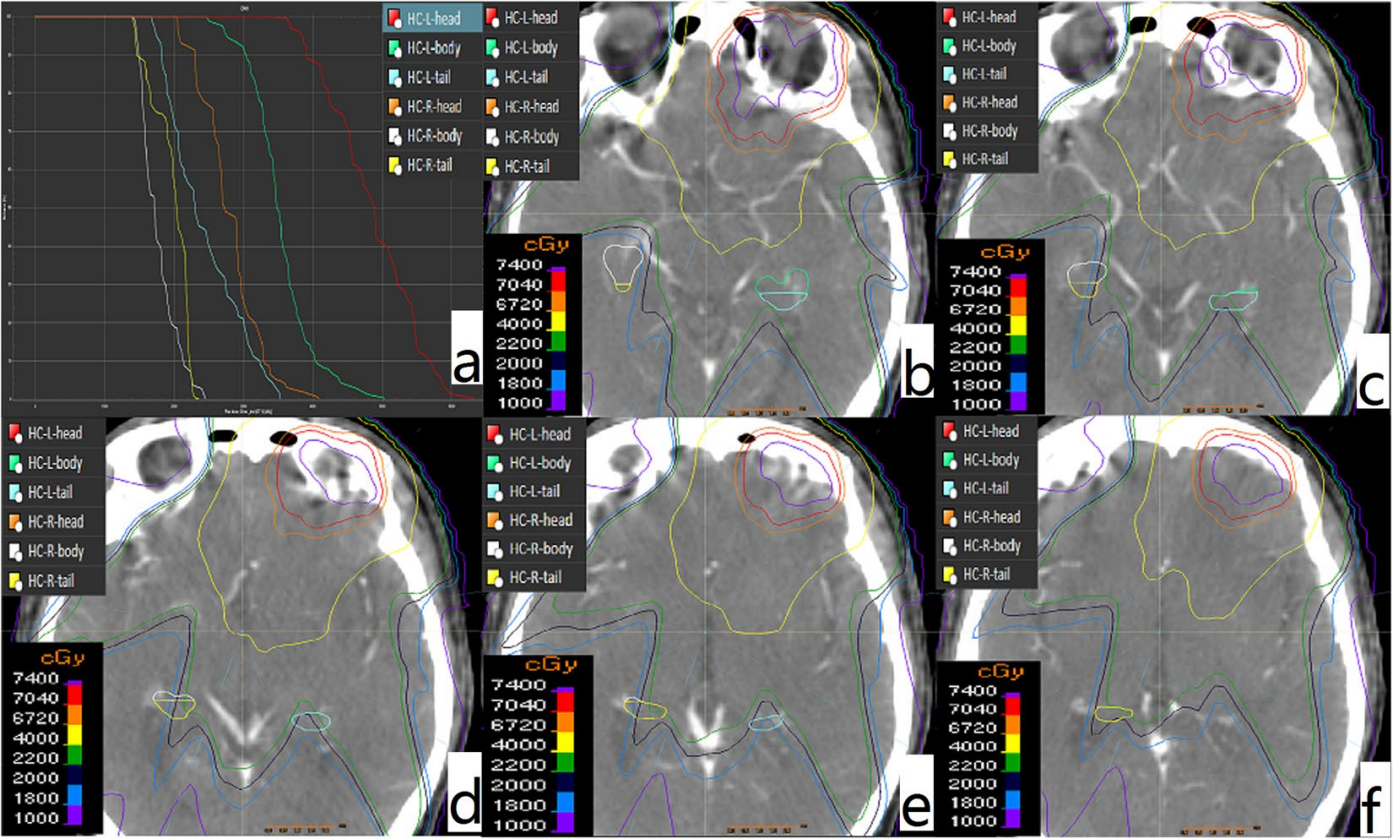
Diagram of the radiation dose of right HT was higher than HB. (**a**) The DVH curve; (**b**–**f**) the dose coverage of right HB and HT.

It is worth noting that the value of HT was greater than HB in V_20_% of HC-R (showed in Table 3). By rechecking the data, we found that there were 2 patients whose radiation dose of HT was greater than that of HB in HC-R. The first patient had extensive invasion lesions involving the skull base, left cavernous sinus, left orbital apex, left optic nerve and left internal and external rectus, and the dose curve was showed in Fig. 3. The other patient was just clivus invasion. This situation may occur randomly because of no HC sparing in the process of radiotherapy planning. It can be seen that because of different extension range, there is possibility that the radiation dose of HT is greater than HB.

IMRT can make HC sparing plan, Han *et al*.^19^ reported that D_mean_ of HC could decreased from (2411 ± 239) cGy to (1414 ± 159) cGy in the IMRT sparing plan. Gu *et al*.^18^ reported that D_mean_ of HC could decrease from 1518 cGy to 899 cGy and 896 cGy respectively under technology of VMRT and IMRT sparing plan. Our results showed that under no HC sparing plan, the radiation dose of HH, HB and HT decreased in turn. We can infer that the radiation dose of the HH, HB and HT will also decrease in HC sparing plan. Compared with HB and HT, HH is closer to positions such as skull base, cavernous sinus etc that was easily invaded in locally advanced NPC. Therefore special attention should be paid to HH that was irradiated with higher dose.

Because of the anatomical characteristics of HC, the anterior-inferior part of HC is close to nasopharynx, while the posterior-superior part of HC is away from nasopharynx. It can be assumed that the radiation dose of HH, HB and HT was different. It is relatively difficult to segment HC into HH, HB and HT, and some method is difficult to implement^22^. This small sample study adopted a simple method which was relatively easy to complete the segmentation. As far as we know, we described the radiation dose of HH, HB and HT for the first time. We confirmed that the radiation dose of HH, HB and HT was different, and the radiation dose of HH, HB and HT decreased in turn. In future we can find more reliable and operable demarcation method, and expand the sample size for further dose study. Further improve the cognitive evaluation of neurological function and explore the roles of HH, HB and HT in cognitive function damage caused by radiotherapy.
